# Antimicrobial Activity of *Bacillus Persicus* 24-DSM Isolated from Dead Sea Mud

**DOI:** 10.2174/1874285801711010372

**Published:** 2017-12-29

**Authors:** Nehaya Al-Karablieh

**Affiliations:** Hamdi mango center for scientific research, The University of Jordan, Amman, Jordan

**Keywords:** Dead Sea mud, *Bacillus persicusi*, Antimicrobial activity, *Corynebacterium diphtheria*, Antagonistic assay, Inhibitory concentration

## Abstract

**Intorduction:**

Dead Sea is a hypersaline lake with 34% salinity, gains its name due to the absence of any living macroscopic creatures. Despite the extreme hypersaline environment, it is a unique ecosystem for various halophilic microorganisms adapted to this environment.

**Aims & Objectives:**

Halophilic microorganisms are known for various potential biotechnological applications, the purpose of the current research is isolation and screening of halophilic bacteria from Dead Sea mud for potential antimicrobial applications.

**Methods & Materials:**

Screening for antagonistic bacteria was conducted by bacterial isolation from Dead Sea mud samples and agar plate antagonistic assay. The potential antagonistic isolates were subjected to biochemical characterization and identification by 16S-rRNA sequencing. Among the collected isolates, four isolates showed potential antagonistic activity against *Bacillus subtilis* 6633 and *Escherichia coli* 8739. The most active isolate (24-DSM) was subjected for antagonistic activity and minimal inhibitory concentration against different gram positive and negative bacterial strains after cultivation in different salt concentration media. Results: The results of 16S-rRNA analysis revealed that 24-DSM is very closely related to *Bacillus persicus* strain B48, which was isolated from hypersaline lake in Iran.

**Conclusion:**

Therefore, the isolate 24-DSM is assigned as a new strain of *B. persicusi* isolated from the Dead Sea mud. *B. persicusi* 24-DSM showed higher antimicrobial activity, when it was cultivated with saline medium, against all tested bacterial strains, where the most sensitive bacterial strain was *Corynebacterium diphtheria* 51696.

## 1 INTRODUCTION

Dead Sea is a hypersaline lake with 34% salinity, gains its name due to the absence of any living macroscopic creatures. The lake consists of deeper northern basin and shallow southern basin which is recently dried up and used for commercial mineral production.

The water level is dependent on the balance between amount of freshwater inflow and evaporation [1]. Jordan River is the main source of freshwater inflow, in addition to several water springs and the complex system of underwater springs, which is recently discovered [2].

The Dead Sea is an increasingly extreme environment due to continuous evaporation, UV radiation, high temperature and salinity [3, 4]. Na+ precipitates as halite while the salts of Mg2+ are more soluble. The present ionic composition is 2M Mg2+, 1.5M Na+, 0.5M Ca2+, 0.2M K+, 6.5M Cl-, 0.1M Br-, which inhibits most of the living forms [5]. Another mineral-rich constituent of the Dead Sea is the mud. The therapeutic effect of processed Dead Sea mud is related to its high content of minerals [6]. It has been demonstrated that Dead Sea salts and mud are useful in treating skin disorders and skin diseases such as psoriasis [7], dermatitis [8].

Despite the extreme hypersaline environment, the Dead Sea is a unique ecosystem harbouring the various halophilic microorganisms adapted to its environment such as; unicellular green algae (*Dunaliella*), archaea (*Haloferax*, *Haloarcula*, *Halobaculum* and *Halorubrum*) and bacteria (*Halomonas*, *Chromohalobacter*, *Salibacillus, Arthrobacter*, *Kocuria*, *Vibrio*, *Salinivibrio*, *Erythrobacter* and *Bacillus*) [9, 10].

Halophilic microorganisms are known for various potential biotechnological applications such as; pigment production, hydrocarbon degradation, Polyhydroxy alkanoate production, halocin production, exopolysaccharide production, bioemulsifier production and halotolerant enzyme production [11, 12], most of these products are salt stable and carry out reactions efficiently under extreme conditions [13]. Different antibiotics are now less effective due to bacterial resistant; therefore the demand for stable and efficient antibiotics is increasing worldwide. The present study focuses on isolation and screening of halophilic bacteria from Dead Sea mud for potential antimicrobial applications.

## 2 MATERIALS AND METHODS

### 2.1 Bacterial Strains and Cultivation Condition

Bacterial strains used in this study are listed in Table **1**. The used bacterial strains were cultivated on Luria Broth medium (LB) as a complex medium (10g tryptone, 5g yeast extract, 10g NaCl, 15g agar (Bio Basic, Canada) for 1L, pH 7.5), or Mannitol-Glutamate medium (MG) as a minimal medium for *Pseudomonas* (10g mannitol, 2g L-Glutamic acid, 0.5g KH_2_PO_4_, 0.2g NaCl, 0.2g MgSO_4_.7H_2_O, 15g agar (Bio Basic, Canada) for 1 L, pH 7.0). Bacterial isolates obtained from the Dead Sea mud were cultivated in Marine Broth (MB) medium (10g tryptone, 5g yeast extract, 15g agar (Bio Basic, Canada) for 1L, pH 7.5) prepared by diluted Dead Sea water (salinity 3.2%).

### 2.2 Sampling and Bacterial Isolation form Dead Sea Mud

**Fig. (1):**
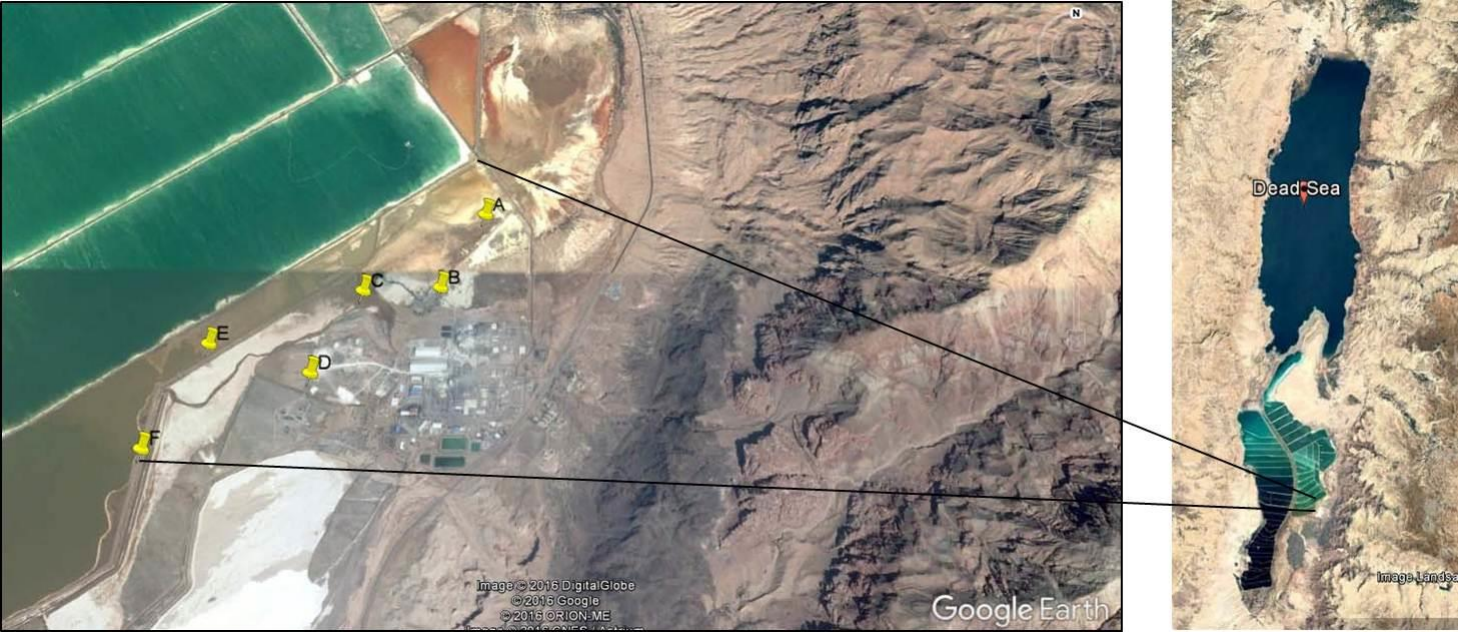
Map of Dead Sea showing six sampling sites: A, B, C, D, E and F.

Dead Sea mud samples (6) were collected in August 2014, the hottest month in Jordan, from shore close to Arab Potash Company in Jordan, where high salinity level in the mud due to the activity of the company in minerals harvesting from the Dead Sea (Fig. **1**), geographic coordinates are shown in Table **2**. Mud temperature, salinity and pH were measured in suit by a portable conductivity and pH meter (Ohause, China).

The mud samples were used for bacterial isolation on MB, briefly, ten grams of Dead Sea mud were mixed with 90ml sterile Dead Sea water, incubated for 1h at 37°C, 250rpm, followed by a series of 10-fold dilution with sterile Dead Sea water, one hundred microliters of each dilution were spread on MB and incubated at 37°C for 24h. After incubation, colonies were counted for viable bacterial cells from each sample separately as CFU/ml. Single colonies were selected for further uses and cryopreservation in 15% Glycerol.

### 2.3 Screening for Antagonistic Isolates

Preliminary screening for antagonistic isolates was conducted by agar plate assay based on described protocol by [14]. The obtained isolates were used as antagonistic isolates against *B. subtilis* 6633 and *E. coli* 8739. The bacterial strains and isolates were grown on appropriate medium at 37°C for 24h, re-suspended in sterile 0.9% NaCl to an optical density (OD) _600_ ~1.0 (approximately 10^7^ CFU/ml), one hundred microliters of *B. subtilis* and *E. coli* were seeded separately on LB plates, after drying, ten microliters of bacterial isolates were applied separately on the inoculated plates and incubated at 37°C for 24h. Ten microliter of 25mg/ml of Chloramphenicol (Cm) and 10µl of sterile 0.9% NaCl were used as positive and negative control respectively. The plates were examined for growth inhibition zones caused by the isolates by visual inspection (Table **3**).

### 2.4 Identification and Biochemical Characterization

Four active isolates against *E. coli* 8739 and *B. subtilis* 6633, namely; 1-DSM, 5-DSM, 7-DSM and 24-DSM, were subjected to gram staining, biochemical characterization by API (Analytical profile index) 50CHB and API-E (BioMérieux, France) according to the manufacturer’s instructions [15, 16] Table **4**, and 16S-rRNA amplification using the forward (5’-AGAGTTTGATCCTGGCTCAG-3’) and reverse (5’-TACGG(CT)TACCTTGTTACGCTT-3’) primers [17]. In a total volume of 50µl, the reaction was performed as previously described by [18]. The PCR amplicons were sequenced at Macrogen Inc., South Korea. To determine the phylogenetic affiliation, sequences were initially compared to the available databases by using nucleotide blast search at NCBI BLAST Website (https://blast.ncbi.nlm.nih.gov/Blast.cgi).

**Fig. (2):**
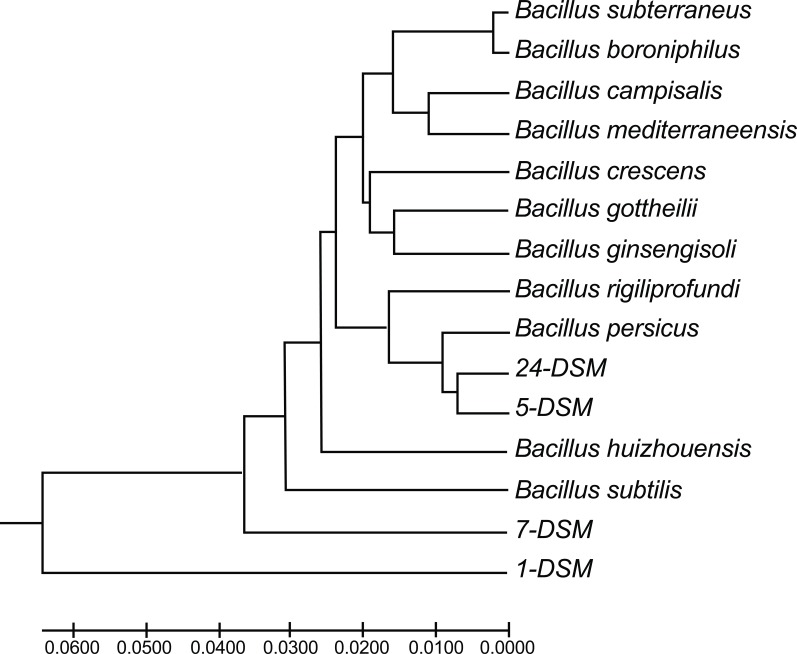
Phylogenetic tree of the Dead Sea mud isolates and their close relatives based on 16S-rRNA. The sequences were retrieved from NCBI database. The evolutionary distances were computed using the Maximum Composite Likelihood method [19] and evolutionary analyses were conducted in MEGA7 [18].

Sequences of the closest relative were retrieved from the database and used to construct a phylogenetic tree using MEGA7 [19], by the Maximum Composite Likelihood method [20] (Fig. **2**). The accession numbers of the 16S-rRNA used for comparison were: NR_041275.1 *B. boroniphilus*; NR_042274.1 *B. foraminis*; NR_104749.1 *B. subterraneus*; NR_108491.1 *B. gottheilii*; NR_109068.1 *B. ginsengisoli*; NR_109140.1 *B. persicus* strain B48; NR_133974.1 *B. huizhouensis*; NR_135898.1 *B. rigiliprofundi*; NR_137360.1 *B. campisalis*; NR_144741.1 *B. mediterraneensis*; NR_136792 *B. crescens* strain JC247 and AB018486 *B. subtilis* 6633. The GC contents of the sequences were calculated by Oligo Calculator (http://mbcf149.dfci.harvard.edu/docs/oligocalc.html) (Table **5**).

### 2.5 *In Vitro* Growth of 24-DSM

**Fig. (3):**
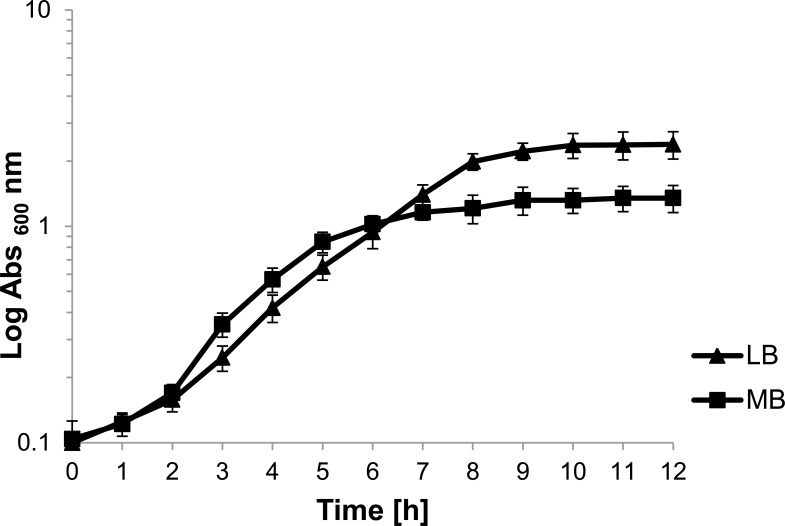
*In vitro* growth of 24-DSM in LB and MB medium at 37°C as determined by measurement of the OD_600_. Data represent the means of three independent cultures ± standard deviation.

The most active isolate (24-DSM) was chosen for further study. In order to study salinity influence on fitness of 24-DSM, *in vitro* growth experiments were conducted. The isolate was cultured in LB and MB broth media at 37°C and 250rpm. The OD_600_ was monitored hourly until the culture had entered the late stationary phase. Three independent replicates were used for each medium, with ratio 1:5 of liquid medium to air (Fig. **3**).

### 2.6 Antimicrobial Assay and MIC Determination

**Fig. (4):**
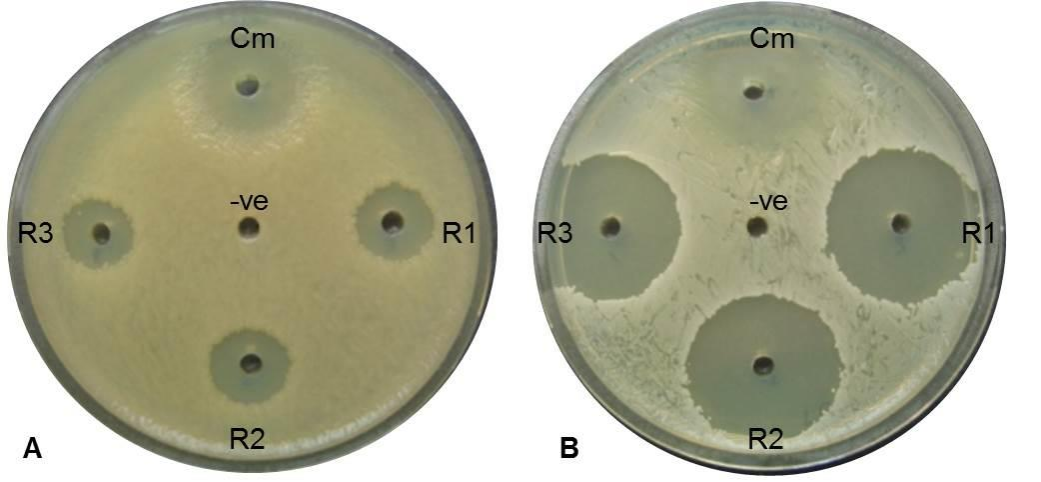
Antagonistic assay against different bacterial strains by the 10X concentrated 24-DSM supernatants; as a representative samples A: *Escherichia coli* 6633, B: *Corynebacterium diphtheria* 51696. Bacterial strains suspension (100 µl of OD_600_ ~1.0) were spread on MHB agar medium, after drying, 10 µl of 10X concentrated 24-DSM supernatants were loaded in 4mm in diameters well (R1, R2 and R3), 10 µl of 12.5 mg/ml of chloramphenicol was used as positive control (Cm) and 10 µl of 10X desalted medium was used as a negative control (-ve).

Antimicrobial activity of 24-DSM was conducted by agar plate well diffusion assay, against 8 human pathogens Table (**6**), four of them were gram negative bacterial strains and 4 of them were gram positive. Active fresh culture of bacterial strains re-suspended in sterile 0.9% NaCl to an OD_600_ ~1.0, one hundred microliters of bacterial suspension were seeded on Muller Hinton agar (Bio Basic, Canada) plates separately. The 24-DSM was cultivated in LB and MB liquid medium at 37°C and 250rpm, the cultures were cultivated at late exponential phase OD_600_ ~1 and OD_600_ ~2 in MB and LB medium respectively, centrifuged at 4000rpm. The supernatant were filtered by 0.22µm filter for removal of bacterial cells, desalted by XAD-4 resin (Sigma-Aldrich, USA), and concentrated 10 times (10X) by vacuum concentrator (Eppendorf, Germany). Three independent cultures were used for each medium as replicates. Ten microliters of the 10X supernatants were loaded in 4mm in diameter well, chloramphenicol was used as a positive control (10µl of 25mg/ml), and 10µl of 10X desalted medium was used as a negative control. The inoculated plates were incubated at 37°C for 48h, growth inhibition zones were monitored by visual inspection (Fig. **4**). The influence of medium used for 24-DSM cultivation has been analyzed by ANOVA and least significant differences at P = 0.05 using SPSS Statistics 21.

The MIC of 10X supernatants was determined by a two-fold dilution assay in Mueller-Hinton broth (MHB) medium (Bio Basic, Canada). All tests were done in triplicates according to the National Center for Clinical Laboratory Standards recommendations [21]. The Bacterial strains were incubated at 37°C, MHB was used as a blank and MHB inoculated with test strains was used as growth control. Bacterial growth was examined visually after 24h of incubation. The MIC was considered as the lowest volume of 10X supernatants that completely stopped visible cell growth, in general, differences in MIC values were considered significant if they were at least four-fold, this cut-off is consistent with previous publications [22].

## 3 RESULTS

### 3.1 Physiological and Microbiological Properties of the Samples

The mud samples were highly saline with a range of 40-45%, the pH values of the samples were 5.7 to 6.4 and relatively high in suit temperature with the range 37 to 40. These results indicate the high acidic salinity of the mud samples. In spite of these harsh conditions, the viable bacterial counts in the samples were relatively high ranging from 6.83 X 10^3^ to 2.53 X 10^5^ (Table **3**).

### 3.2 Screening for Antagonistic Isolates

Twenty-Four different isolates were chosen based on collection site and colony morphology to be used in preliminary screening for antagonistic isolates. Ten different isolates were able to form inhibition zones on plates inoculated separately with *E. coli* 8739 and *B. subtilis* 6633. But only, four of these isolates were able to inhibit growth of both *E. coli* 8739 and *B. subtilis* 6633, namely; 1-DSM, 5-DSM, 7-DSM and 24-DSM, where inhibition zones ranged from around 7.5 to 9 mm in diameter in case of *E. coli* 8739 and from around 3.5 to 12 mm in diameter in case of *B. subtilis* 6633. Another 4 isolates were able to inhibit *E. coli* 8739 growth only and 2 isolates were able to inhibit *B. subtilis* 6633 growth only (Table **3**).

### 3.3 Identification and Biochemical Characterization

In this study, the four isolates which were able to inhibit both *E. coli* 8739 as a gram negative model organism and *B. subtilis* 6633 as a gram positive model organism were identified based on gram staining, biochemical characterization and 16S rRNA analysis. The four isolates were gram positive and had had the same biochemical properties Table **4**. For instance, all possess catalase, oxidase and urease enzymes, able to produce Indole and acetone, reduce nitrates, hydrolyze gelatin and utilize the same set of carbon compounds. These biochemical properties were all shared by the reference strain *B. subtilis* 6633. Analysis of the 16S rRNA revealed that the four isolates were found to be very close to *Bacillus* species with identity 96-98%. The closest relative to each isolates is shown in Table **5** and Fig. (**2**). The 16S rRNA sequences revealed a relatively high GC content (up to 55%). Nucleotide sequence accession number for 24-DSM generated in this study has been deposited in GenBank under accession number MF037224.

### 3.4 *In vitro* Growth of 24-DSM

**Fig. (5):**
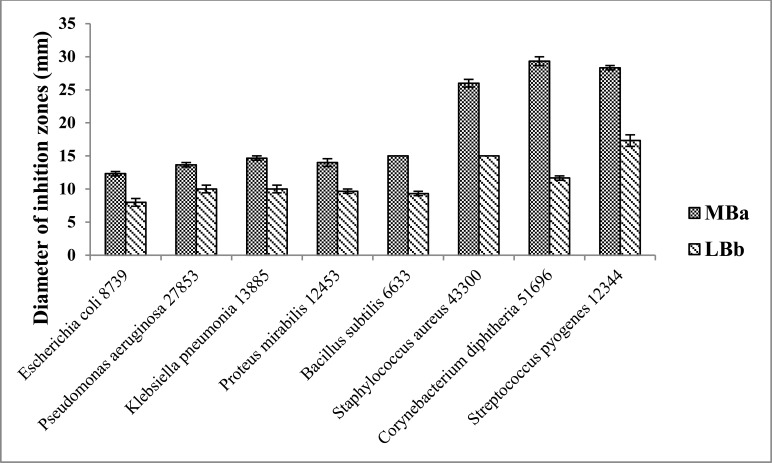
Susceptibility of different bacterial strains to 10X concentrated 24-DSM supernatants by agar plate diffusion assay. Average of three replicates ± standard error. Bacterial strains suspension (100 µl of OD_600_ ~1.0) were spread on MHB agar medium, after drying, 10 µl of 10X concentrated 24-DSM supernatants were loaded in 4mm in diameters well. a,b: ANOVA and least significant differences at P = 0.05 using SPSS Statistics 21 for cultivation medium.

In order to test the salinity effect on overall fitness of 24-DSM (the most active isolate), *in vitro* growth experiments were conducted. The isolate was cultured in LB and MB at 37°C. The OD_600_ was monitored continuously until cultures had entered the late stationary phase (Fig. **5**). Growth of 24-DSM was generally faster in MB than that in LB with doubling times of ~60 min in MB and ~90 min in LB. The culture reached the stationary phase within 6h with an OD_600_ of ~1 in MB, while in LB the stationary phase was reached at OD_600_ of ~2. These results demonstrate that the salinity contributes to *in vitro* fitness of 24-DSM.

### 3.5 Antimicrobial Assay and MIC Determination

Susceptibility of different gram negative and gram positive bacterial strains toward 24-DSM was tested by agar plate diffusion assay and MIC determination using the desalted-10X concentrated-supernatants of 24-DSM cultivated separately in MB and LB media (Table **5**). The results showed that the desalted-10X concentrated-supernatants of 24-DSM that cultivated in both media were able to inhibit the tested bacterial strains. The Diameters of inhibition zones caused by 24-DSM cultivated in MB (range from ~12 to ~29 mm) were significantly higher than that caused by 24-DSM cultivated in LB (range from ~8 to ~17 mm). In contrast to the MIC values, there was no significant influence for the used cultivation medium on the values of MIC, except for *C. diphtheria* 51696, since the MIC value of the desalted-10X concentrated-supernatants of 24-DSM cultivated in MB is 8-fold lower than that cultivated in LB medium, if we consider at least 4-fold difference is a significant difference. These results indicate that the salinity in MB medium might enhance the antimicrobial activity of 24-DSM mainly against *C. diphtheria* 51696.

It was remarkable to mention that, in both cultivation media, the gram positive bacterial strains were more susceptible to the desalted-10X concentrated-supernatants of 24-DSM than gram negative bacterial strains as shown in Table **6**.

## 4 DISCUSSION

The physicochemical properties of the Dead Sea mud samples were analyzed. The salinity percentage was found to be very high (up to 45%) and the pH was found to be acidic (up to 6.4), these are typical properties of Dead Sea water, which making the Dead Sea as the most well-known hypersaline environment [23, 24]. In spite of these harsh conditions, the number of viable bacterial cells in the tested mud samples were very high, reached 2.5 X 10^5^ CFU/ml, in comparison to the bacterial cell count in open sea marine water, where it reaches up 3 X 10^6^ bacterial cell/ml [25], despite that these number was obtained by using unspecific fluorescent staining techniques [26].

Many studies have proved that the black, hypersaline Dead Sea mud is useful in treating skin disorders and diseases. Therefore, the mud has been extensively used in mud packs, masks, topical body and facial treatments in spas surrounding the lake, and as a base for the preparation of soaps, creams, and ointments for skin care marketed worldwide [6]. Little is known about the microbiological aspects of the Dead Sea mud. Therefore, this research was conducted to expand our knowledge about bioactive halophilic bacteria living in such hypersaline conditions.

Halophilic bacteria have been previously isolated from Dead Sea Cost, Jordan [27] and Dead Sea water [10]. In this study, twenty four different isolates were isolated from form Dead Sea mud (Data not shown).

In the preliminary screening for antimicrobial properties of these isolates, ten of these isolates showed antimicrobial activity. But only, four isolates showed antimicrobial activity against both model bacterial strains *Escherichia coli* 8739 and *Bacillus subtilis* 6633, namely, 1-DSM, 5-DSM, 7-DSM and 24-DSM. These isolates were subjected for further identification, they were gram positive bacteria, it was reported that gram positive bacteria have adapted to environmental stress such as high salinity through anionic phospholipids in the membrane composition [28, 29]. These four isolates belong to the genus *Bacillus* which is mostly saprophytes, commonly found in soil; a few are animal, especially insect, parasites or pathogens [30].

The isolates characterized by high GC content up to 55%, these results are in agreement with Jacob results (2012), where he suggested that the thick cell wall, high peptidoglycan content, high GC content and resistant-spore formation are the main reasons for the survival of *Bacillus* in such a harsh condition of high salinity [10]. The newly characterized isolates possess catalase and oxidase enzymes like *B. halosaccharovorans* sp. Nov., which was isolated from hypersaline lake [31], and showed *Bacillus* profile, for some enzymatic activity and carbohydrate metabolism, that suggested by [15].

*Bacillus* isolates described in this study belong to *Bacillus crescens* and *Bacillus persicus,* these species were encountered in salty environment [32, 33]. The 1-DSM and 7-DSM are very close related (96% identity) to *B. crescens* strain JC247 isolated from soil samples collected from Rann of Kutch, Gujarat, India [33] and 5-DSM and 24-DSM are very similar (98% identity) to *B. persicus* strain B48, which is a novel species isolated from hypersaline lake in Iran [32]. The 24-DSM and 5-DSM have been clustered with *B. persicus* in the phylogenetic tree based on 16S rRNA sequences. Therefore, the isolate 24-DSM is assigned as new strain of *B. persicusi* isolated from the Dead Sea mud.

Growth on different NaCl concentration is used to distinguish *Bacillus* species [31]. In this study, the salinity effects on overall fitness of 24-DSM and its antimicrobial activity have been investigated. Although, 24-DSM growth was faster in MB (3.2% salinity) than in LB (1% salinity), it was not able to reach high population. In spite of these, 24-DSM antimicrobial activity was higher, when it was cultivated in higher saline. This significant effect was clearer against gram positive bacteria, mainly; *C. diphtheria*, followed by *S. pyogenes* and *S. aureus*, by using agar plate diffusion assay, but this significance has disappeared when MIC technique was used except in *C. diphtheria*, where the difference in MIC value was 8-fold.

These results are in parallel with other results, the susceptibility of *C. diphtheria* to a wide range of antimicrobial agents has been demonstrated [34]. In contrast with other results where *S. pyogenes* has been found to be resistance to some macrolides but susceptible to clindamycin and miocamycin [35] and meticillin-resistant *S. aureus* has been reported as an emerging threat [36].

The antimicrobial properties of Dead Sea mud were related to the chemical and physical properties of the mud [37], it has been recently reported that halophilic microorganisms from the Dead Sea could be used in biotechnological application such as extracellular protease production, Polyhydroxy alkanoates production, halocin production and bio-emulsifier production [12], Zinc and Copper removal [38], Lead and Cadmium removal [39]. In this study, isolation of halophilic microorganisms from the Dead Sea mud for antimicrobial application was reported. Further investigation will be conducted for the identification of potential antimicrobial compound.

## CONCLUSION

Four *Bacillus* isolates were isolated from Dead Sea mud, the antimicrobial activity was observed for these isolates. The most active isolate in antimicrobial activity was assigned as *Bacillus persicus* 24-DSM and it was found to be active against some gram positive and negative bacterial strains. This finding might open the door for utilizing halophilic microorganisms found in the Dead Sea in new antibiotics production.

## Figures and Tables

**Table 1:** Bacterial strains used in this study.

**Bacterial strains**	**ATCC no.**
**Gram Negative**	
*Escherichia coli *	8739
*Pseudomonas aeruginosa *	27853
*Klebsiella pneumonia *	13885
*Proteus mirabilis *	12453
**Gram Positive**	
*Bacillus subtilis *	6633
*Staphylococcus aureus *	43300
*Corynebacterium diphtheria *	51696
*Streptococcus pyogenes *	12344

**Table 2:** Geographic coordinates of the sampling sites, physiochemical properties of the mud and viable bacterial count of the samples.

**Sampling site**	**Coordinates**	**Salinity (%)**	**Temperature (°C)**	**pH**	**CFU/ml**
**A**	31° 6'15.78"N 35°30'56.90"E	40	38	6.1	3.46 X 10^5^
**B**	31° 5'58.04"N 35°30'46.13"E	45	40	5.7	2.53 X 10^5^
**C**	31° 5'57.33"N 35°30'26.79"E	41	37	6.4	1.46 X 10^5^
**D**	31° 5'38.56"N 35°30'15.52"E	42	39	5.9	6.83 X 10^3^
**E**	31° 5'45.14"N 35°29'49.91"E	40	38	6.2	3.56 X 10^5^
**F**	31° 5'22.42"N 35°29'37.43"E	43	37	5.7	6.66 X 10^4^

**Table 3:** Preliminary screening for antagonistic activity of Dead Sea mud isolates against *E. coli *8739 and *B. subtilis. *

–	Diameter of Inhibition zones (mm^*^)	
**Isolate nr.**	***Escherichia Coli* 8739**	***Bacillus Subtilis *6633**
**1-DSM**	**8.33 ± 0.67**	**3.67 ± 2.66**
**5-DSM**	**7.66 ± 0.37**	**10.33 ± 1.45**
**7-DSM**	**7.66 ± 0.67**	**9.67 ± 0.33**
10-DSM	0.00 ± 0.00	6.00 ± 1.15
12-DSM	4.66 ± 0.00	0.00 ± 0.00
14-DSM	4.67 ± 0.00	0.00 ± 0.00
18-DSM	5.00 ± 0.00	0.00 ± 0.00
20-DSM	0.00 ± 0.00	6.33 ± 1.45
21-DSM	4.67 ± 0.00	0.00 ± 0.00
**24-DSM**	**9.00 ± 0.00**	**12.33 ± 0.45**
Cm	10.00 ± 0.60	26.00 ± 0.40
0.9% NaCl	0.00 ± 0.00	0.00 ± 0.00

*Average of three replicates ± standard error of means, 10µl of OD600 ~1.0 isolates suspension spotted on plate previously seeded with bacterial strains; 10µl of 25mg/ml chloramphenicol (Cm) and 10µl of 0.9% NaCl were used as positive and negative control respectively.

**Table 4:** Some physiological properties of Dad Sea mud bacterial isolates.

**Characteristics**	***B. subtilis* 6633**	**1-DSM **	**5-DSM**	**7-DSM**	**24-DSM**
Gram staining	+	+	+	+	+
Catalase	+	+	+	+	+
Oxidase	+	+	+	+	+
**API 20E tests**					
Ortho NitroPhenyl-ßDGalactopyranosidase	-	-	-	-	-
Arginine dihydrolase	-	-	-	-	-
Lysine decarboxylase	-	-	-	-	-
Ornithine decarboxylase	+	+	+	+	+
Citrate utilization	-	-	-	-	-
H_2_S production	-	-	-	-	-
Urease	+	+	+	+	+
Tryptophane deaminase	-	-	-	-	-
Indole production	+	+	+	+	+
Acetoin production	+	+	+	+	+
Gelatinase	+	+	+	+	+
NO2 production	+	+	+	+	+
**API 50CHB tests**					
Glycerol	+	+	+	+	+
Erythritol	-	-	-	-	-
D-Arabinose	-	-	-	-	-
L-Arabinose	+	+	+	+	+
Ribose	+	+	+	+	+
D-Xylose	+	+	+	+	+
L-Xylose	-	-	-	-	-
Adonitol	-	-	-	-	-
β-Methylxyloside	-	-	-	-	-
Galactose	-	-	-	-	-
D-Glucose	+	+	+	+	+
D-Fructose	+	+	+	+	+
D-Mannose	+	+	+	+	+
L-Sorbose	-	-	-	-	-
Rhamnose	-	-	-	-	-
Dulcitol	-	-	-	-	-
Inositol	+	+	+	+	+
Mannitol	+	+	+	+	+
Sorbitol	+	+	+	+	+
α-Methyl-Dmannoside	-	-	-	-	-
α-Methyl-D-glucoside	+	+	+	+	+
N-Acetylglucosamine	-	-	-	-	-
Amygdalin	+	+	+	+	+
Arbutin	+	+	+	+	+
Aesculin	+	+	+	+	+
Salicin	+	+	+	+	+
Cellobiose	+	+	+	+	+
Maltose	+	+	+	+	+
Lactose	-	-	-	-	-
Melibiose	-	-	-	-	-
Sucrose	+	+	+	+	+
Trehalose	+	+	+	+	+
Inulin	-	-	-	-	-
Melezitose	-	-	-	-	-
D-Raffinose	-	-	-	-	-
Starch	+	+	+	+	+
Glycogen	+	+	+	+	+
Xylitol	-	-	-	-	-
β-Gentiobiose	+	+	+	+	+
D-Turanose	+	+	+	+	+
D-Lyxose	-	-	-	-	-
D-Tagatose	-	-	-	-	-
D-Fucose	-	-	-	-	-
L-Fucose	-	-	-	-	-
D-Arabitol	-	-	-	-	-
L-Arabitol	-	-	-	-	-
Gluconate	-	-	-	-	-
2-Ketogluconate	-	-	-	-	-
5-Ketogluconate	-	-	-	-	-

+: indicates positive reaction; - : indicates negative reaction.

**Table 5:** Closest relatives of the Dead Sea mud isolates with their identity percentage. Isolates with more than 97% are considered to be the same species.

**Isolate**	**Close relative **	**Identity %**	**GC%**
1-DSM	*Bacillus crescens *strain JC247	96	54
5-DSM	*Bacillus persicus* strain B48	98	54
7-DSM	*Bacillus crescens *strain JC247	96	55
24-DSM	*Bacillus persicus* strain B48	98	55

**Table 6:** MIC determination 10X concentrated 24-DSM supernatants to some bacterial strains.

	MIC* **(µl/ml)**	
**Bacterial strains**	**MB**	**LB**
**Gram Negative**		
*Escherichia coli* 8739	>20	>20
*Pseudomonas aeruginosa* 27853	>20	>20
*Klebsiella pneumonia* 13885	10	>20
*Proteus mirabilis *12453	10	>20
**Gram Positive**		
*Bacillus subtilis *6633	5	10
*Staphylococcus aureus *43300	2.5	5
*Corynebacterium diphtheria 51696*	1.25^a^	10
*Streptococcus pyogenes 12344*	2.5	5

* MIC determination in MHB medium by the dilution assay was repeated three times in each case thereby confirming consistencies of MIC values. Differences in MIC values were only considered significant if they were at least 4-fold.
